# Fatal West Nile Virus Infection After Probable Transfusion-Associated Transmission — Colorado, 2012

**Published:** 2013-08-09

**Authors:** Sharon Kelly, Tuan N. Le, Jennifer A. Brown, Elisabeth W. Lawaczek, Matthew Kuehnert, Ingrid B. Rabe, J. Erin Staples, Marc Fischer

**Affiliations:** Presbyterian/St. Luke’s Medical Center, Denver; Bonfils Blood Center, Denver; Tri-County Health Dept, Greenwood Village; Colorado Dept of Health and Environment; Div of Healthcare Quality Promotion; Div of Vector-Borne Diseases, National Center for Emerging and Zoonotic Infectious Diseases, CDC

West Nile virus (WNV) is transmitted to humans primarily by infected mosquitoes. However, WNV also can be transmitted through infected blood products or solid organs (1). Since 2003, the U.S. blood supply has been routinely screened for WNV RNA. The Food and Drug Administration (FDA) recommends that blood collection agencies perform WNV nucleic acid testing (NAT) year-round on all blood donations, either in minipools of six or 16 donations (MP-NAT) or as individual donations (ID-NAT) (2). Since implementation of screening, 12 transfusion-associated transmissions of WNV have been documented (3–5). This report describes a case of fatal WNV encephalitis in an immunosuppressed patient after probable transfusion-associated transmission. The implicated donation was reactive by MP-NAT but nonreactive by ID-NAT on routine screening and was released for transfusion. During the subsequent investigation, retrospective testing of the donated unit showed discrepant ID-NAT results and evidence of WNV-specific immunoglobulin M (IgM) and neutralizing antibodies. Although WNV is rarely transmitted through screened blood products, clinicians should consider WNV disease in patients with compatible symptoms who were recently transfused. Further evaluation is required to determine the relative risks and benefits of different strategies to manage MP-NAT–reactive minipools when all constituent donations are nonreactive by subsequent ID-NAT

## Case Report

In August 2012, a man with non-Hodgkin’s lymphoma was admitted to hospital for chemotherapy and autologous stem cell transplant. His cancer was in partial remission after six cycles of chemotherapy. He had been screened for subclinical infections 14 days before stem cell collection, and all testing, including WNV NAT, was negative. Stem cells were collected 8 days before admission and transplanted on hospital days 8 and 9. The patient developed gastrointestinal symptoms on hospital day 18, followed by fever and hypotension on hospital day 28. On hospital day 29, he developed altered mental status, somnolence, and respiratory failure; sedation and mechanical ventilation precluded a full neurologic assessment. Cerebrospinal fluid (CSF) collected on hospital day 30 showed an elevated glucose (103 mg/dL [normal: 40–80 mg/dL]) and normal protein (44 mg/dL [normal: 5–50 mg/dL]) with two white blood cells/mm^3^ (normal: 0–5/mm^3^) and 46 red blood cells/mm^3^ (normal: 0–5/mm^3^). This CSF sample was negative for bacteria by Gram and acid-fast stains, for cryptococcal antigen by latex agglutination, and for cytomegalovirus, BK virus, herpes simplex virus, JC virus, human herpes virus 6, and varicella-zoster virus by polymerase chain reaction (PCR); however, the sample was not tested for WNV. Magnetic resonance imaging showed meningeal and cortical changes consistent with inflammation, and electroencephalogram findings were consistent with diffuse encephalopathy. The patient’s mental status did not improve after discontinuation of sedation; support was withdrawn, and he died on hospital day 47. Postmortem evaluation showed diffuse encephalitis. WNV IgM antibodies were identified in serum collected on hospital day 43. WNV RNA was detected by TaqMan reverse transcription-PCR (RT-PCR) on brain and spinal cord tissues collected at autopsy.

## Public Health Investigation

The patient was hospitalized continuously for 4 weeks before illness onset without known outdoor exposure; therefore, mosquito-borne WNV transmission was unlikely. A public health investigation was conducted to determine the source of his WNV infection and prevent further transmission by removal of any contaminated blood products. The patient had received allogenic, leukoreduced, irradiated blood products during hospital days 13–30, including 6 units of red blood cells, 2 units of platelets, and 2 units of fresh frozen plasma. He received 2 additional units each of red blood cells and platelets on day 44.

The hospital blood bank investigated the blood products received before hospital day 43, when the patient’s first WNV-positive serum was collected. WNV serologic testing was conducted on all 10 associated donors. Nine donors had no evidence of WNV infection by WNV IgM enzyme-linked immunosorbent assay. One apheresis donor was positive for WNV IgM antibodies and for WNV neutralizing antibodies by plaque reduction neutralization testing performed on serum collected 56 days after the implicated donation. This donor reported no history of illness before or after donation and no travel, but did report outdoor exposure with mosquito bites. The apheresis platelet unit was donated on the recipient’s hospital day 9 and transfused on day 14. The implicated donation was initially screened as part of a minipool with five other donations; the MP-NAT was reactive using the Cobas TaqScreen West Nile Virus Test (Roche Molecular Systems). However, all of the units comprising the minipool were nonreactive when tested by ID-NAT using the same assay and were released into the blood supply per FDA guidance at the time of the investigation (2).

In addition to the implicated unit of platelets, fresh frozen plasma was also derived from the original donation but was not transfused. The quarantined unit of fresh frozen plasma was retrieved and tested for WNV. Freshly thawed aliquots from this unit were reactive for WNV on four of five replicate Cobas TaqScreen West Nile Virus Tests, but WNV RNA could not be detected by conventional Taqman RT-PCR. An aliquot from the same plasma unit, tested 58 days after being thawed and stored at 39.2°F (4.0°C) was nonreactive by the Procleix WNV NAT assay (Gen-Probe Incorporated). The unit was positive for WNV IgM antibodies by microsphere-based immunosorbent assay, WNV immunoglobulin G antibodies by enzyme-linked immunosorbent assay, and WNV neutralizing antibodies by plaque reduction neutralization testing.

Per FDA guidelines, the donor was deferred from making further donations for 120 days after the implicated donation. The donor had made seven donations of platelets and plasma in the 120 days before the implicated donation, and one donation of platelets and plasma 35 days after the implicated donation. The blood products from those donations had been transfused to nine recipients. Four of these recipients had since died; however, their deaths were thought to be related to causes other than WNV infection because none had discernible signs or symptoms of WNV disease. However, no clinical samples were available for testing. The five surviving recipients were monitored clinically and remained asymptomatic but were not tested for WNV infection.

### Editorial Note

This report describes a fatal case of WNV disease after probable transfusion-associated transmission via platelets collected from an asymptomatic infected blood donor. Although transfusion-transmitted WNV has been reported after initial screening was nonreactive by MP-NAT, it has not been documented after screening was nonreactive by ID-NAT. This case demonstrates the potential for transmission of WNV through transfusion of blood products that screen nonreactive by ID-NAT, despite having detectable WNV-specific IgM and neutralizing antibodies.

What is already known on this topic?

Blood collection agencies in the United States routinely screen donors for West Nile virus (WNV) RNA using minipool nucleic acid testing (MP-NAT) augmented by individual donation testing (ID-NAT). Since 2003, approximately 3,500 reactive units have been removed from the blood supply, preventing transfusion transmission of WNV. This system is effective; however, 12 transfusion-associated cases have been identified since implementation of WNV NAT screening.

What is added by this report?

This report describes the first probable case of transfusion-associated WNV infection in which the donation was reactive by MP-NAT but nonreactive by ID-NAT on initial screening. The case occurred in an immunosuppressed patient. Virus-specific immunoglobulin M and neutralizing antibodies could be detected in the implicated donation. These findings are suggestive that transmission occurred from a blood donation with a low concentration of WNV even in the presence of presumptively protective WNV-specific antibodies.

What are the implications for public health practice?

Clinicians should maintain a high index of suspicion for cases of transfusion-associated WNV disease, especially in immunosuppressed patients with compatible symptoms who have recently received blood products. Further evaluation is required to determine the relative risks and benefits of different strategies to manage MP-NAT–reactive minipools when all constituent donations are nonreactive by subsequent ID-NAT.

Persons infected with WNV typically develop a transient viremia that occurs 1–2 weeks after infection and lasts approximately 7 days. Viremia, detectable by NAT, is present before symptom onset and early in the course of illness, but usually resolves as the patient develops WNV antibodies. FDA recommends that blood collection agencies screening for WNV by MP-NAT establish criteria to switch to ID-NAT, which is more sensitive than MP-NAT, during periods of high WNV activity in their collection areas (2). At the time of the implicated donation, the blood testing agency had not reached its threshold for conversion to ID-NAT. During initial screening, the implicated donation was reactive by MP-NAT. ID-NAT was performed to identify the infected donation(s) in the minipool; however, none of the constituent donations were reactive, so the products were released into the blood supply. The blood collection and testing agencies involved have now decided to discard all constituent units of reactive minipools when an ID-NAT reactive donation cannot be identified.

It is likely that this transfusion-associated transmission occurred because the donor had a waning viremia that was sufficiently low to be inconsistently identified by either MP-NAT or ID-NAT. NATs performed during initial screening and during retrospective testing of the plasma co-component from the implicated donation yielded discrepant results. The Cobas TaqScreen West Nile Virus Test and Procleix WNV NAT assays typically detect lower levels of WNV RNA than conventional RT-PCR. However, at very low levels of viremia, NAT assays performed on minipools or individual units can exhibit test-to-test variability and might require replicate testing to detect viral RNA (6–8). In addition, the nonreactive Procleix NAT might reflect lower WNV RNA concentration in the sample because of viral degradation during prolonged storage at 39.2°F (4.0°C).

In published reports of transfusion-associated transmissions of WNV, IgM could not be detected in implicated donations when stored donor segments or co-components were available for testing (1,3). However, researchers at a conference in 2003 reported detection of WNV IgM in one of 13 WNV RNA-positive plasma co-components identified during multiple transfusion-transmission investigations during that year; neutralizing antibody results were not reported (9). An in vitro study using serum from blood donors containing both WNV RNA and IgM antibodies showed that 10 (36%) of 28 of these specimens could infect Vero cells (10). In the case described in this report, WNV transmission appears to have occurred despite the presence of detectable WNV-specific IgM and neutralizing antibodies in the implicated donation. The protective benefit of these antibodies might have been insufficient to prevent infection in this severely immunosuppressed patient.

Since the implementation of routine screening of blood products for WNV, transfusion-associated WNV infections have been rare. However, clinicians should consider WNV disease in any patient with compatible symptoms who has received a blood transfusion during the 28 days before illness onset. This is particularly important in immunosuppressed patients, such as stem cell recipients, who might be more susceptible to WNV infection at very low viral concentrations in the transfused blood product and, once infected, are at greater risk for developing WNV neurologic disease. Suspected cases should be reported immediately to hospital blood banks and public health authorities to facilitate prompt investigation and quarantine of potentially infectious blood products. Further evaluation is required to determine the relative risks and benefits of different strategies to manage MP-NAT-reactive minipools when all constituent donations are nonreactive by subsequent ID-NAT.
